# Orientin promotes diabetic wounds healing by suppressing ferroptosis via activation of the Nrf2/GPX4 pathway

**DOI:** 10.1002/fsn3.4360

**Published:** 2024-07-27

**Authors:** Jia‐yi Yang, Chen Zhuang, Yu‐zhe Lin, Yi‐tian Yu, Chen‐cheng Zhou, Chao‐yang Zhang, Zi‐teng Zhu, Cheng‐jie Qian, Yi‐nan Zhou, Wen‐hao Zheng, Yu Zhao, Chen Jin, Zong‐yi Wu

**Affiliations:** ^1^ Department of Gynaecology The Second Affiliated Hospital and Yuying Children's Hospital of Wenzhou Medical University Wenzhou Zhejiang China; ^2^ The Third Peoples Hospital of Ouhai District Wenzhou Zhejiang China; ^3^ Alberta Institute, Wenzhou Medical University Wenzhou Zhejiang China; ^4^ Department of Orthopaedics The Second Affiliated Hospital and Yuying Children's Hospital of Wenzhou Medical University Wenzhou Zhejiang China; ^5^ Key Laboratory of Orthopaedics of Zhejiang Province Wenzhou Zhejiang China; ^6^ The First School of Medicine Wenzhou Medical University Wenzhou Zhejiang China; ^7^ The Second School of Medicine Wenzhou Medical University Wenzhou Zhejiang China

**Keywords:** diabetes wound, ferroptosis, mitochondrial dysfunction, Nrf2/GPX4 signaling pathway, Orientin

## Abstract

Diabetic patients often experience delayed wound healing due to impaired functioning of human umbilical vein endothelial cells (HUVECs) under high glucose (HG) conditions. This is because HG conditions trigger uncontrolled lipid peroxidation, leading to iron‐dependent ferroptosis, which is caused by glucolipotoxicity. However, natural flavonoid compound Orientin (Ori) possesses anti‐inflammatory bioactive properties and is a promising treatment for a range of diseases. The current study aimed to investigate the function and mechanism of Ori in HG‐mediated ferroptosis. A diabetic wound model was established in mice by intraperitoneal injection of streptozotocin (STZ), and HUVECs were cultured under HG to create an in vitro diabetic environment. The results demonstrated that Ori inhibited HG‐mediated ferroptosis, reducing levels of malondialdehyde (MDA), lipid peroxidation, and mitochondrial reactive oxygen species (mtROS), while increasing decreased levels of malondialdehyde, lipid peroxidation, and mitochondrial reactive oxygen species, as well as increased levels of glutathione (GSH). Ori treatment also improved the wound expression of glutathione peroxidase 4 (GPX4) and angiogenesis markers, reversing the delayed wound healing caused by diabetes mellitus (DM). Additional investigations into the mechanism revealed that Ori may stimulate the nuclear factor‐erythroid 2‐related factor 2 (Nrf2)/GPX4 signaling pathway. Silencing Nrf2 in HG‐cultured HUVECs negated the beneficial impact mediated by Ori. By stimulating the Nrf2/GPX4 signaling pathway, Ori may expedite diabetic wound healing by decreasing ferroptosis.

## Graphical abstract

It appears that Orientin (Ori) offers protective effects in human umbilical vein endothelial cells (HUVECs) by enhancing the activity of mitochondria and reducing ferroptosis, which is triggered by high glucose (HG) levels. Furthermore, it is anticipated that the stimulation of the nuclear factor‐erythroid 2‐related factor 2 (Nrf2)/GPX4 signaling pathway may improve the process of wound healing in diabetic patients who are undergoing Orientin therapy.

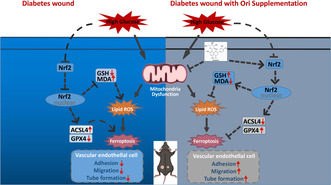

## 1 INTRODUCTION

Patients with diabetes are at a greater risk of developing disabilities and passing away as a result of issues associated to wounds on the skin that do not heal in the appropriate manner (Bian et al., 2020; Umar et al., 2022). Although there have been notable improvements in managing blood glucose levels, treating wounds, and removing dead tissue through surgery, diabetic wounds continue to present a large and urgent problem in the medical field globally. This issue places a considerable strain on society and the economy (Wang et al., 2022). People who have diabetes have a more difficult time healing wounds because they produce more reactive oxygen species (ROS) and inflammatory cytokines than people who do not have diabetes (Feng, Yu, et al., 2022; Gao et al., 2021). High glucose (HG) levels trigger endothelial ROS overproduction, leading to endothelial dysfunction (Viollet et al., 2012). Recent research has also explored that the metabolism of glucolipids influences ferroptosis (Feng, Yin, et al., 2022; Huang et al., 2023).

Ferroptosis is a recently found variation of programmed cell death that takes place as a result of the uncontrolled oxidation of lipids. This particular form of cell death is unique to individuals who are deficient in iron (Han et al., 2022). Ferroptosis exhibits unique biological traits in comparison to other types of cell demise, including intracellular iron surplus and heightened ROS levels, which ultimately result in lipid peroxidation (Huang et al., 2022). Ferrostatin‐1 (Fer‐1), a low‐molecular‐weight medication, effectively inhibits ferroptosis by scavenging lipid peroxides (Luo et al., 2022). Studies have shown that ferroptosis affects not only cancer treatment outcomes, but also the results of metabolic diseases, neurodegenerative disorders, cardiomyopathies, ischemia–reperfusion injuries, and the healing of wounds caused by diabetes (Irimia‐Dominguez et al., 2020; Zhao et al., 2022). The healing process of diabetic wounds involves mitochondrial dysfunction and increased ROS levels, and ferroptosis could significantly influence the pathophysiology of diabetic wounds (Schwank‐Xu et al., 2021). However, the interplay between human umbilical vein endothelial cells (HUVECs) and ferroptosis in the diabetic microenvironment has not been thoroughly investigated by researchers. Therefore, targeting ferroptosis can be a promising approach to treating diabetic wounds and other diabetes‐related conditions.

Activation of the gene nuclear factor‐erythroid 2 related factor 2 (Nrf2), which is responsible for the oxidative stress response, has been proposed as a potential method to prevent the occurrence of ferroptosis (Anandhan et al., 2023; Park et al., 2019). Nrf2, an essential controller of ferroptotic cell demise, has demonstrated its ability to regulate several genes, including the glutathione peroxidase 4 (GPX4) gene (Ge et al., 2021). A study that was carried out by Li and Liang, along with other researchers, indicated that the activation of the Nrf2/GPX4 signaling pathway has the ability to effectively reduce ferroptosis (Li, Hu, et al., 2023; Xing et al., 2023). These noteworthy findings were reported in the journal *Cell* (Liang et al., 2023). Additionally, the Nrf2/GPX4 signaling pathway is an essential component in the maintenance of the functionality and stability of mitochondrial activities. As a result, we hypothesized that the Nrf2/GPX4 signaling pathway is closely connected to the process of wound healing in diabetic patients.

The C‐glycosyl flavonoid known as Orientin is found in fenugreek, and it possesses a wide range of bioactivities and therapeutic benefits, including anti‐inflammatory, antidiabetic, anti‐oxidative, and autophagy‐inducing properties (An et al., 2015; Jing et al., 2020). Molecular biology research has shown that Orientin has a closely relationship with oxidative stress, mitochondrial function, and the Nrf2 signaling pathway (Xia et al., 2023; Xiao et al., 2018). On the other hand, there is a dearth of research that attempts to investigate the influence of Orientin on the recuperation of diabetic wounds and the mechanisms that are related with it. The purpose of this work was to evaluate the effects of Orientin on HUVECs that have been subjected to HG, as well as the processes that are responsible for these effects and the positive effects that Orientin has on a mouse wound model of diabetes. According to the findings, Orientin was able to successfully inhibit ferroptosis by reducing oxidative stress, enhancing mitochondrial activity, and regulating the Nrf2 signaling pathway. These findings imply that Orientin may be a feasible therapy option for diabetic wounds.

## 2 MATERIALS AND METHODS

### 2.1 Ethics statements

This research was allowed to continue by the Institutional Animal Care and Use Committee at Wenzhou Medical University, which gave us permission to move further. Following the guidelines outlined in the National Institutes of Health Guide for the Care and Use of Laboratory Animals, the surgical operations, treatments, and postoperative animal care protocols were carried out.

### 2.2 Reagents and antibodies

From MedChemExpress (MCE) in Monmouth Junction, NJ, USA, Orientin (HY‐N0405, 99.02% purity), Erastin (a ferroptosis inducer), and Ferrostatin‐1 (a selective ferroptosis inhibitor) were obtained. The antibodies used in this study were sourced from Abcam (Cambridge, UK) for acyl‐CoA synthetase 4 (ACSL4) (ab155282, 1:1000), GPX4 (ab252833, 1:1000), MFN1 (ab221661, 1:1000), MFN2 (ab205236, 1:1000), and glyceraldehyde 3‐phosphate dehydrogenase (GAPDH) (9485, 1:3000). Proteintech Group, which is situated in Chicago, Illinois, United States of America, was the source of the primary antibodies that targeted Nrf2 (16396‐1‐AP, 1:1000), CD31 (or platelet endothelial cell adhesion molecule‐1, PECAM‐1) (11265‐1‐AP, 1:1000), vascular endothelial growth factor (VEGF polyclonal antibody (VEGF‐A) (19003‐1‐AP, 1:1000), and Lamin B (12987‐1‐AP, 1:2000). In addition, the acquisition of 4′,6‐diamidino‐2‐phenylindole (DAPI) was carried out by Beyotime, which is located in Shanghai, China. The research utilized a wide variety of chemicals and their respective origins. These included the 5‐ethynyl‐2′‐deoxyuridine (EdU) assay, which was obtained from Elabscience (Wuhan), C11‐BODIPY, which was obtained from Invitrogen, FerroOrange, which was obtained from Tongren (Beijing), phosphate‐buffered saline (PBS), fetal bovine serum (FBS), 0.25% trypsin, and Dulbecco's modified Eagle's medium (DMEM) were obtained from Gibco (New York). Sigma, located in St. Louis, was the source of all of the extra chemicals that were utilized in the inquiry.

### 2.3 Cell culture

American Type Culture Collection (ATCC), which is located in Manassas, Virginia, was the source of the HUVECs that were collected. The cells were cultivated in Dulbecco's modified Eagle's medium (DMEM) that contained 10% heat‐inactivated fetal bovine serum (FBS), 1% penicillin, and 1% streptomycin. The cells were then maintained in an incubator at a temperature of 37°C and a carbon dioxide (CO_2_) content of 5%. Newly cultured cells were placed in T‐75 cell culture flasks (Falcon) at a density of 2500 to 3000 cells/cm^2^. Every 48 h, the medium was switched out. Using a light microscope, we could see that the cultures had reached confluence after 6 or 7 days.

### 2.4 Cell viability assay

In the current investigation, the vitality of HUVECs was evaluated with the assistance of a CCK‐8 (Cell Counting Kit‐8) assay kit that was obtained from Elabscience, which is located in Wuhan, China. After completing the treatment that was given, the cells were treated with a CCK‐8 working solution that included 10% CCK‐8 reagent. This allowed the cells to continue producing the reagent for a period of 1–2 days. After this period of time had passed, the cells were placed in an incubator that had been preheated to 37°C for a period of 1 h. Through the utilization of a microplate reader that was made by Thermo Fisher Scientific and located in Waltham, Massachusetts, United States of America, absorbance measurements were performed on each individual well; 450 nm was the wavelength at which the measurements were carried out.

### 2.5 EdU staining assay

The EdU Cell Proliferation Kit, manufactured by Beyotime in China, was utilized to facilitate the process of cell proliferation in accordance with the directions provided by the manufacturer. HUVECs were exposed to EdU for 4 h, fixed with 4% polyformaldehyde for 30 min, and then stained with Hoechst 33342 for nuclear staining. The cells that had been stained were observed and captured using a microscope.

### 2.6 C11‐BODIPY and FerroOrange staining

Using C11‐BODIPY (Invitrogen, USA), we were able to determine the levels of lipid peroxidation that were present in the cells. Following the completion of the treatment, the HUVECs were subjected to a working solution of C11‐BODIPY at a concentration of 1 mol L1 for a period of 30 min. An investigation on the presence of Fe^2+^ within the cell was carried out with the assistance of a FerroOrange probe that was produced by Dojindo in Shanghai, China. After being washed with a PBS solution, HUVECs were subjected to a washing procedure for 30 min before being exposed to a FerroOrange working solution at a concentration of 1 mol/L. A confocal scanning microscope that was created by Nikon, a firm located in Japan, was utilized in order to study the cells that had been tagged.

### 2.7 Quantification of MDA and GSH activities

After undergoing two washings with a solution of PBS, the HUVECs were then put through a lysis process on ice for a period of 30 min. The levels of MDA and GSH activity in each sample were determined by using assay kits supplied by Sigma (St. Louis, Missouri, United States of America) that were available for commercial use and following the instructions that were provided by the supplier.

### 2.8 Western blot analysis

The Western blot analysis was carried out in a manner that was consistent with the protocol that was outlined in the section that was cited before this one. HUVECs were lysed using phosphatase and protease inhibitors at a concentration of 1 mM from Sigma‐Aldrich. The lysis solution used was radio‐immunoprecipitation assay (RIPA) buffer, which was purchased from the Beyotime Institute of Biotechnology. In order to prepare the cells for lysis, they were washed with ice‐cold PBS for a period of 30 min at a temperature of 4°C. It was necessary to expose the cell lysates to centrifugation at a force of 12,000 *g* in order to achieve the goal of protein separation. The sodium dodecyl sulfate–polyacrylamide gel electrophoresis (SDS–PAGE) procedure was carried out with 20 g of the protein that had been isolated. After being incubated for a considerable amount of time at a temperature of 4°C, the proteins were then transferred to a membrane made of polyvinylidene difluoride (PVDF). After being washed three times with a Tris‐buffered saline solution that contained 0.1% Tween 20 (TBST), the membranes were then subjected to secondary antibodies that were conjugated with horseradish peroxidase (HRP) and specifically bound to the primary antibodies. For a period of 4 h, this incubation procedure was carried out at room temperature throughout the duration. Tween 20 solution at a concentration of 0.1% was used to perform a pretreatment on the membranes before this operation was carried out. An enhanced chemiluminescence (ECL) reagent was utilized in conjunction with the ChemiDoc XRS+ system, which was manufactured by Bio‐Rad and located in Hercules, California, United States of America. The most recent version of the Bio‐Rad Image Lab Software was utilized in order to perform quantitative analysis on the protein bands produced.

### 2.9 Transmission electron microscopy (TEM)

Under the microscope, the microstructure of the cell was investigated. Following a single night of fixation at a temperature of 4°C, the cells were then subjected to a postfixation procedure that lasted for 30 min and utilized a 1% solution of osmium tetroxide (OSO_4_). Following that, an embedding procedure and a series of ethanol rinses were carried out in order. This was followed by the creation of thin sections, which were then treated with uranyl acetate and lead citrate afterwards. After that, a Hitachi field‐emission optical microscope was used to analyze them.

### 2.10 ROS measurement

For the purpose of determining the levels of reactive oxygen species (ROS) that were present within cells, we utilized the dihydroethidium (DHE) probe that was manufactured by Yeasen Biotechnology, which is located in Shanghai, China. After selecting fields at random, a fluorescence microscope was used to examine the results of the experiment.

### 2.11 Mitochondrial function assays

MitoSox and JC‐1 fluorescent probes were applied in the research project in order to determine the levels of reactive oxygen species and mitochondrial membrane potential (MMP), respectively. For the purpose of capturing images of the regions of interest, fluorescence microscopy was utilized. The outcomes illustrated that mitochondria with elevated membrane potentials, indicating healthy conditions, emitted red fluorescence, while those with decreased membrane potentials, indicating unhealthy conditions, emitted green fluorescence. The aforementioned metrics were obtained using a Nikon microscope manufactured in Japan.

### 2.12 Tube formation assay

The generation of HUVEC tubes on a chamber glass slide coating was accomplished by the use of matrix coagulation procedures. The ECMatrix gel solution was applied to the u‐slide plate, and then the plate was put through an incubation process that lasted for 1 h at a temperature of 37°C. This was done in order to solidify the matrix. During the process, there were three iterations. The pretreated HUVECs were collected by employing trypsin and ethylenediaminetetraacetic acid (EDTA). After the HUVECs were treated, they were placed on the Matrigel that had been processed beforehand. The tube was then formed by placing the dish in an incubator at a temperature of 37°C for a period of 6 h. This was done in order to construct the tube. The tube creation was initially viewed with a phase contrast microscope, and its size was calculated afterwards by quantitatively analyzing a fraction of the surface area of each well.

### 2.13 Cell‐matrix adhesion assay

On a six‐well plate, a HUVEC adhesion assay was performed. The HUVECs were treated with Orientin, HG, and Fer‐1 in the same manner as described earlier. Fibronectin was pre‐coated on the plate at a 5 g/mL concentration for 1 h at 37°C. A uniform quantity of retrieved cells was subsequently cultured at a density of 1 × 10^4^ cells/mL on every coated plate, which was thereafter subjected to incubation for a duration of 30 min. Next, cells that had not adhered to the plate were washed with PBS and then immobilized with 4% paraformaldehyde (PFA). DAPI staining was utilized in order to confirm the presence of adhering cells. A quantitative analysis of cell adhesion was carried out in three distinct places within each well. These locations were chosen because they were considered to be representative of the entire well.

### 2.14 Cell migration assay

To assess HUVECs migration, we employed a Costar polycarbonate membrane Boyden chamber insert with an 8‐μm pore size manufactured in Cambridge, Massachusetts, United States of America. Following the procedures that were described earlier, the insert was positioned inside of a transwell system, and the cells were subjected to treatment with Orientin, HG, and Fer‐1. The cells were then separated from one another, centrifuged to reduce their size, and then resuspended. Following this, a total of 1 × 10^4^ cells were introduced into a transwell apparatus, where they were seeded into a volume of 200 μL of DMEM that did not contain any FBS. Additionally, 700 μL of medium that contained 1% FBS was added to the bottom chamber. Following a 12‐hour incubation period in an incubator containing 5% carbon dioxide, the membrane was fixed with 4% paraformaldehyde and washed three times with PBS. We stained the transwell apparatus with crystal violet and used swabs to remove any accumulated cells on the top surface. Quantification was performed on three arbitrary fields of the individuals that had migrated to the lower surface.

### 2.15 Network pharmacology analysis

In order to collect information on potential pharmacological targets, the Traditional Chinese Medicine Systems Pharmacology Database and Analysis Platform (TCMSP), the Similarity Ensemble Approach (SEA), and SuperPRED were some of the databases that we alluded to in our discussion. In addition, in order to collect DM wound‐related targets, we utilized the Online Mendelian Inheritance in Man (OMIM) database as well as the GeneCard database, as was previously mentioned. The Venn diagrams explain the relationship between Ori and DM wound. Additionally, we were able to identify overlapping targets, which we then chose as common targets for Ori to treat in DM wound.

In the process of constructing a protein–protein interaction (PPI) network, the Search Tool for the Retrieval of Interacting Genes/Proteins (STRING) database is an extremely important component. With the help of the Database for Annotation, Visualization, and Integrated Discovery (DAVID) database, we carried out Gene Ontology (GO) analysis and Kyoto Encyclopedia of Genes and Genomes (KEGG) pathway enrichment analysis in order to acquire a deeper comprehension of the significant biological processes and pathways. Our goal was to provide a more comprehensive explanation of these regions.

### 2.16 Docking analysis

We used ChemBioDraw to draw the Orientin structure and ChemBio3D to minimize the Orientin energy. Universal Protein provided information on the three‐dimensional (3D) structures of the proteins Nrf2 and GPX4. We used PyMOL's “default” parameter choices to produce the docking conformation with the least potential energy. With the help of AutoDock Tools (version 1.5.6), the Protein Data Bank (PDB) file that contained Orientin and the receptor protein was transformed into a PDBQT (Protein Data Bank, Partial Charge (Q), and Atom Type (T)) file. Additionally, the program was utilized to search for active pockets, determine the range of search conformations, and carry out the AutoDock Vina (version 1.2.1) script for the purpose of conducting a protein‐ligand docking investigation. Ultimately, we employed PyMOL, a software developed by the University of California San Francisco (UCSF), to produce the three‐dimensional (3D) visual representation.

### 2.17 siRNA transfection

Invitrogen provided us with the unique small‐interfering RNA (siRNA) associated with the Nrf2 gene, which is based in Carlsbad, California, USA. Transfection of the siRNA into HUVEC cells occurred when the cells reached a confluence of between 30 and 50%. After 12 h, over 95% of the cells remained viable. The cells were cultivated in new medium for a further 3 days before the medium was replaced. This was done before the cells were subjected to any further experiments. The success of the transfection was determined by using Western blot.

### 2.18 STZ‐induced diabetes

After receiving authorization from the Laboratory Animal Ethics Committee and Center at Wenzhou Medical University, experiments were carried out on animals. After being purchased, mice were housed in an environment free of pathogens for a period of 1 week. After that, they were randomly allocated to either the normal group (*n* = 12) or the diabetic group (*n* = 36). In order to induce diabetes, a 100 mg/kg injection of STZ was administered (Jeong et al., 2020; Yuan et al., 2018). The levels of glucose in the blood were measured both before and after the injection, and mice were judged to be hyperglycemic if their levels were greater than 250 mg/dL. We included diabetic mice with levels that were greater than 300 mg/dL.

### 2.19 In vivo wound healing model and drug administration

The study used a total of 48 mice, with 12 mice in each of the Control, DM, DM + Ori, and DM + Fer‐1 groups. The DM mice underwent surgery 2 weeks after STZ injection, with two full‐thickness dermal incisions measuring 0.385 cm^2^ in surface area made on both sides of the dorsal trunk. The wounds had a diameter of 0.7 cm. Following the solubilization of Orientin in dimethyl sulfoxide (DMSO) at a concentration of 50 mg/mL, the protein was subsequently diluted with normal saline. Those in the DM group were given saline, while those in the DM + Ori group were given intraperitoneal injections of the Orientin solution at a dosage of 10 mg/kg on a daily basis. On a daily basis, the Fer‐1 solution was administered to the DM + Fer‐1 group at a dosage of 5 mg/kg, whereas the DM group was given an equivalent volume of saline. The mice were euthanized with pentobarbital sodium on days 5, 10, and 15 after the wound was created. The tissue surrounding the wound was then extracted for histological examination.

### 2.20 Histological analysis

A fixative solution containing 4% paraformaldehyde was used to fix the skin tissues overnight before they were embedded in paraffin. In the subsequent step, the tissues were cut into sections that had a thickness of 4 μm. For the purpose of determining the amount of collagen that was produced, these sections were then stained with hematoxylin and eosin (H&E) as well as Masson's trichrome stain (Beyotime). We examined the sections using an optical microscope and captured photographs.

### 2.21 Tissue immunohistochemical staining

On the seventh day, tissue slices were harvested using the earlier procedure. In the subsequent step, the slices were cooked and treated with a solution that included 10 mM sodium citrate and had a pH of 6.0. After washing, the samples were blocked with 10% FBS for 30 min at room temperature before being analyzed. This was done before beginning the analysis. In the subsequent step, the tissue slices were labeled with alpha smooth muscle actin (α‐SMA) (1:200), CD31 (1:200), and GPX4 (1:200) antibodies. In addition to that, DAPI was used to label the nuclear DNA of the cells. In the end, fluorescent images were obtained by the utilization of the Olympus fluorescence microscope.

### 2.22 Statistical analysis

The mean, together with the standard error of the mean (SEM), was used to describe the continuous data, and it was discovered that the data followed a normal distribution. In order to ensure accuracy, the experiment was repeated at least three times. The analysis of the data was carried out with the assistance of the SPSS software (IBM, New York, United States of America). Tukey's test and one‐way analysis of variance (ANOVA) were utilized in order to evaluate the differences between the groups. It was determined that a p‐value that was lower than 0.05 was statistically significant.

## 3 RESULTS

### 3.1 HG microenvironments induce ferroptosis in HUVECs

**FIGURE 1:**
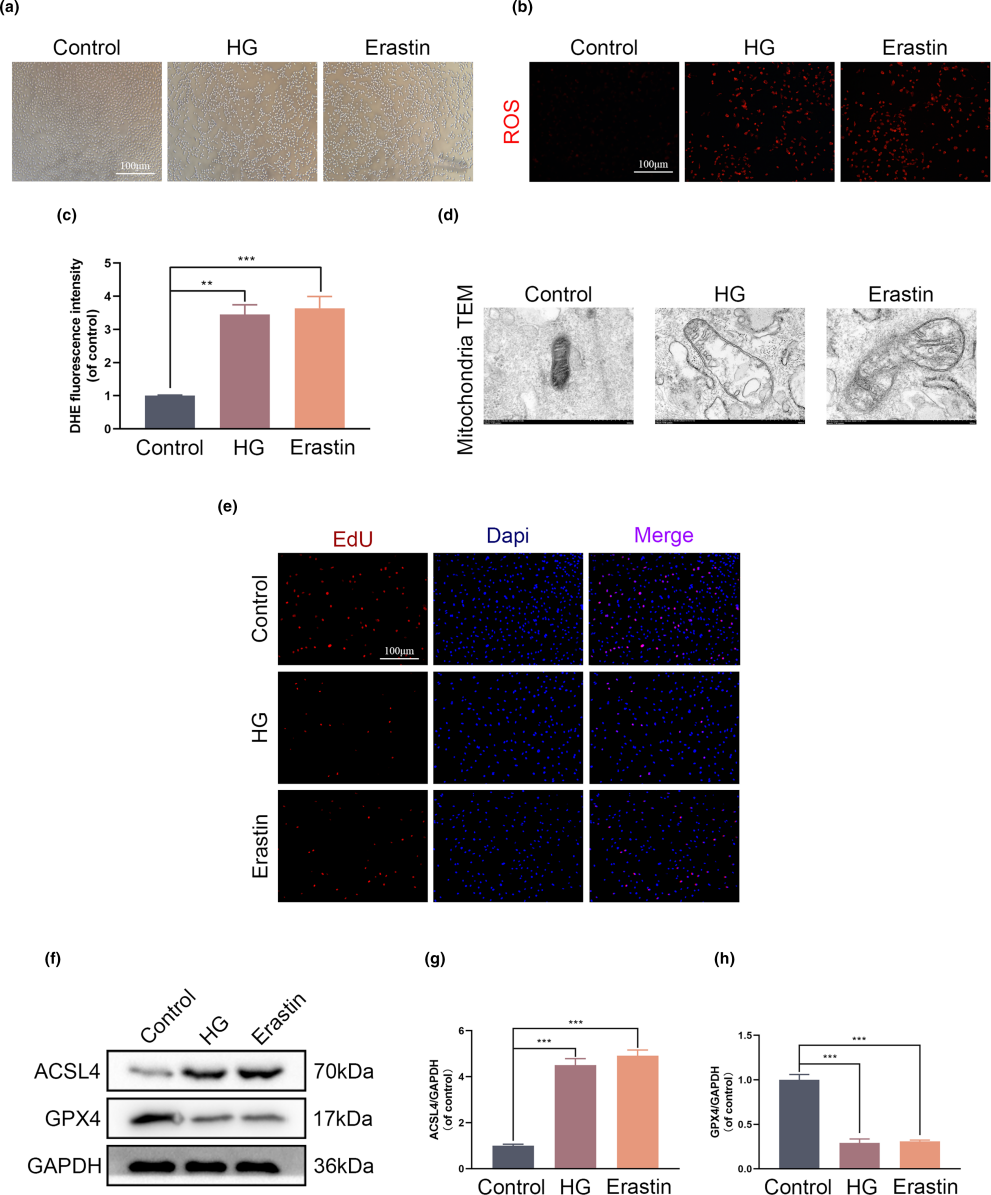
Ferroptosis is induced by high glucose in HUVECs. Human umbilical vein endothelial cells (HUVECs) are given either HG or Erastin for a period of 24 h. (a) Illustrations that are representative of the cell morphology in each of the different groups. (b and c) Quantitative analysis of the DHE staining, along with representative images of each of the different groups. (d) Representative images from the transmission electron microscope of the mitochondria in each of the different groups. (e) Images showing representative examples of the EdU staining in the various groups. (f) A Western blot was used to investigate the levels of ACSL4 and GPX4 expression in HUVECs. A comparison between the two groups is indicated by an asterisk. (g‐h) Western blot quantification analysis of the expression of ACSL4 and GPX4. **p < .01; ***p < .001. All data are from *n* = 3 independent experiments.

In order to replicate the conditions of a diabetic microenvironment, HG was applied to HUVECs at a dosage of 25.5 mM, whereas glucose was applied to control cells at a concentration of 5.5 mM (Li, Rekep, et al., 2023). The study examined the cellular structure and analyzed the effects of HG and Erastin using microscopy. After being exposed to HG and Erastin for a duration of 24 h, the HUVECs in the HG and Erastin groups experienced a morphological change from a spindle shape to a round or rectangular shape, in contrast to the cells in the control group, which maintained their original shape (Figure 1a). These data suggest that the use of HG and Erastin for treatment has negative effects on both the survival and structure of cells. An increase in the fluorescence intensity of DHE as well as an increase in the number of cells that exhibited positive staining for DHE was detected as a consequence of exposure to HG and Erastin (Figure 1b,c). This was observed through the utilization of DHE staining. Through transmission electron microscopy (TEM), it was discovered that the HUVECs that had been treated with HG and Erastin exhibited decreased mitochondrial size and compromised membrane integrity (Figure 1d). When HUVECs were injured, the EdU incorporation test was used to determine whether or not HG and Erastin had any cytoprotective effects on the cells. The findings of our research showed that both HG and Erastin had a significant inhibitory effect on the proliferation of cells as compared to the group that served as the control (Figure 1e). In a nutshell, the administration of HG treatment resulted in a significant reduction in the levels of anti‐ferroptosis proteins GPX4, which was comparable to the effects that were found with the positive control Erastin. In addition, the administration of HG and Erastin both led to a significant increase in the expression of ferroptosis‐associated proteins ACSL4 (Figure 1f–h). The results of this study suggest that ferroptosis plays a role in the cell death that is caused by high glucose levels. This research has the potential to provide novel therapeutic options for the treatment of diabetic wound healing diseases.

### 3.2 Orientin has no cytotoxicity and rescues cell apoptosis induced by HG in HUVECs

**FIGURE 2:**
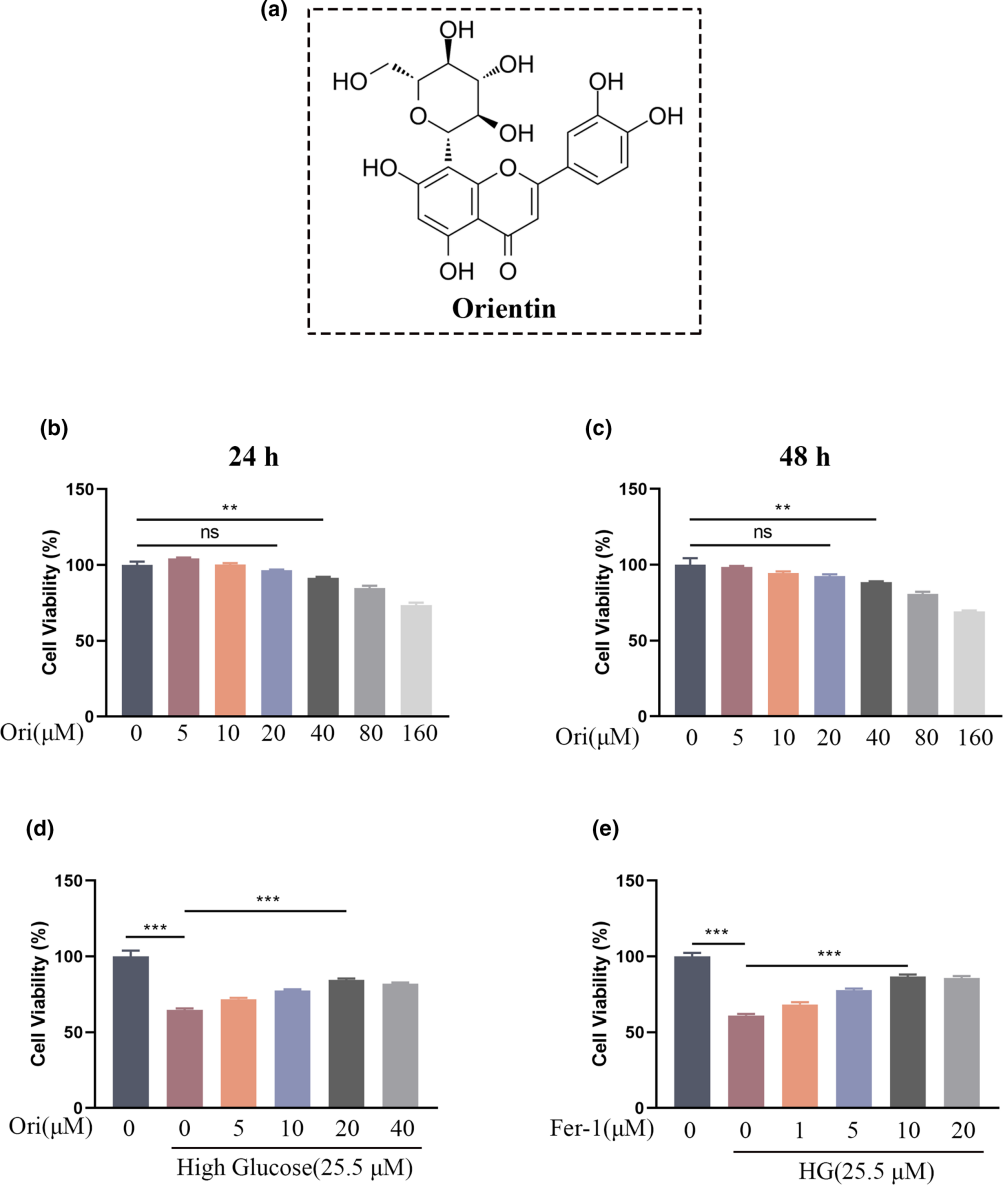
Effects of Orientin on HUVECs viability. (a) Chemical structure of Orientin. (b and c) Percentage of viable cells after treatment with different concentrations of Orientin for 24 or 48 h. (d) Percentage of viable cells after treatment with HG and various concentrations of Orientin. (e) Percentage of viable cells after treatment with HG and various concentrations of Fer‐1. A comparison between the two groups is indicated by an asterisk. **p < .01; ***p < .001; ns = no significance. All data are from *n* = 3 independent experiments.

It is possible to see the molecular structure of Orientin in Figure 2a. HUVECs were subjected to Orientin at a range of concentrations for either 24 or 48 h. According to the findings of our experiment, the highest concentration of Ori that was considered to be safe was 20 μM (Figure 2b,c). When it came to the effect that HG had on the viability of HUVECs, Orientin was found to have positive effect that was dose‐dependent for rescuing HG‐induced HUVECs viability (Figure 2d). To identify the optimal concentration of Fer‐1, ranging from 1 to 20 μM, for rescuing HG‐induced HUVECs, different concentrations were tested (Figure 2e). Subsequent rescue studies employed Fer‐1 at a concentration of 10 μM.

### 3.3 Orientin attenuates ferroptosis caused by HG in HUVECs

**FIGURE 3:**
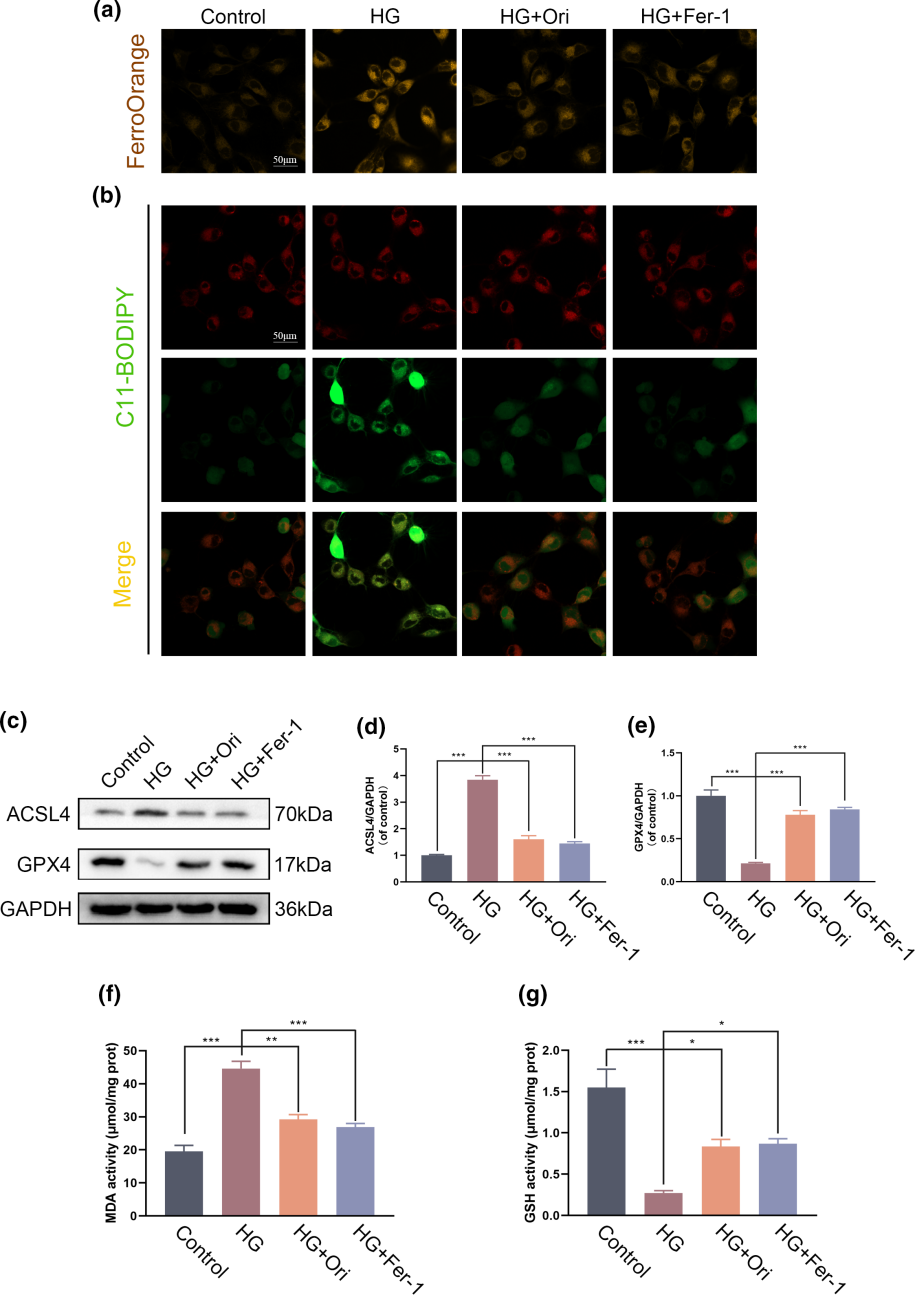
Orientin mitigated HG‐induced ferroptosis in HUVECs. (a) Representative images of FerroOrange staining in different groups. (b) Representative images of C11‐BODIPY staining in different groups. (c–e) Western blot (WB) analysis of the expression of ACSL4 and GPX4 in HUVECs. (f and g) The activities of MDA and GSH in HUVECs were quantitatively determined using MDA and GSH assay kits. A comparison between the two groups is indicated by an asterisk. *p < .05; **p < .01; ***p < .001. All data are from *n* = 3 independent experiments.

After verifying that Orientin did not have any detrimental effects on cell survival or proliferation, we examined its capacity to inhibit HG‐induced ferroptosis. FerroOrange is a probe that specifically detects Fe^2+^. Under the influence of HG stimulation, the fluorescence intensity of the substance rose, but treatment with Orientin and Fer‐1 reversed this effect (Figure 3a). C11‐BODIPY was used to detect intracellular lipid peroxides. In response to HG stimulation, C11‐BODIPY staining revealed a high level of lipid peroxidation, which was reversed by treatment with Orientin and Fer‐1 (Figure 3b). Western blot analysis showed that the expression of ACSL4, a protein associated with ferroptosis, increased in the HG groups, while the expression of GPX4, a protein associated with anti‐ferroptosis, decreased in these groups. However, posttreatment with Orientin and Fer‐1 reversed these changes (Figure 3c–e). MDA and GSH levels reflect the amount of lipid ROS, which significantly increased and decreased due to HG, respectively. However, posttreatment with Orientin and Fer‐1 prevented the oxidative damage caused by HG (Figure 3f,g). Overall, these findings suggest that Orientin may inhibit HG‐induced ferroptosis.

### 3.4 Orientin mitigates mitochondrial dysfunction in HG‐induced HUVECs

**FIGURE 4:**
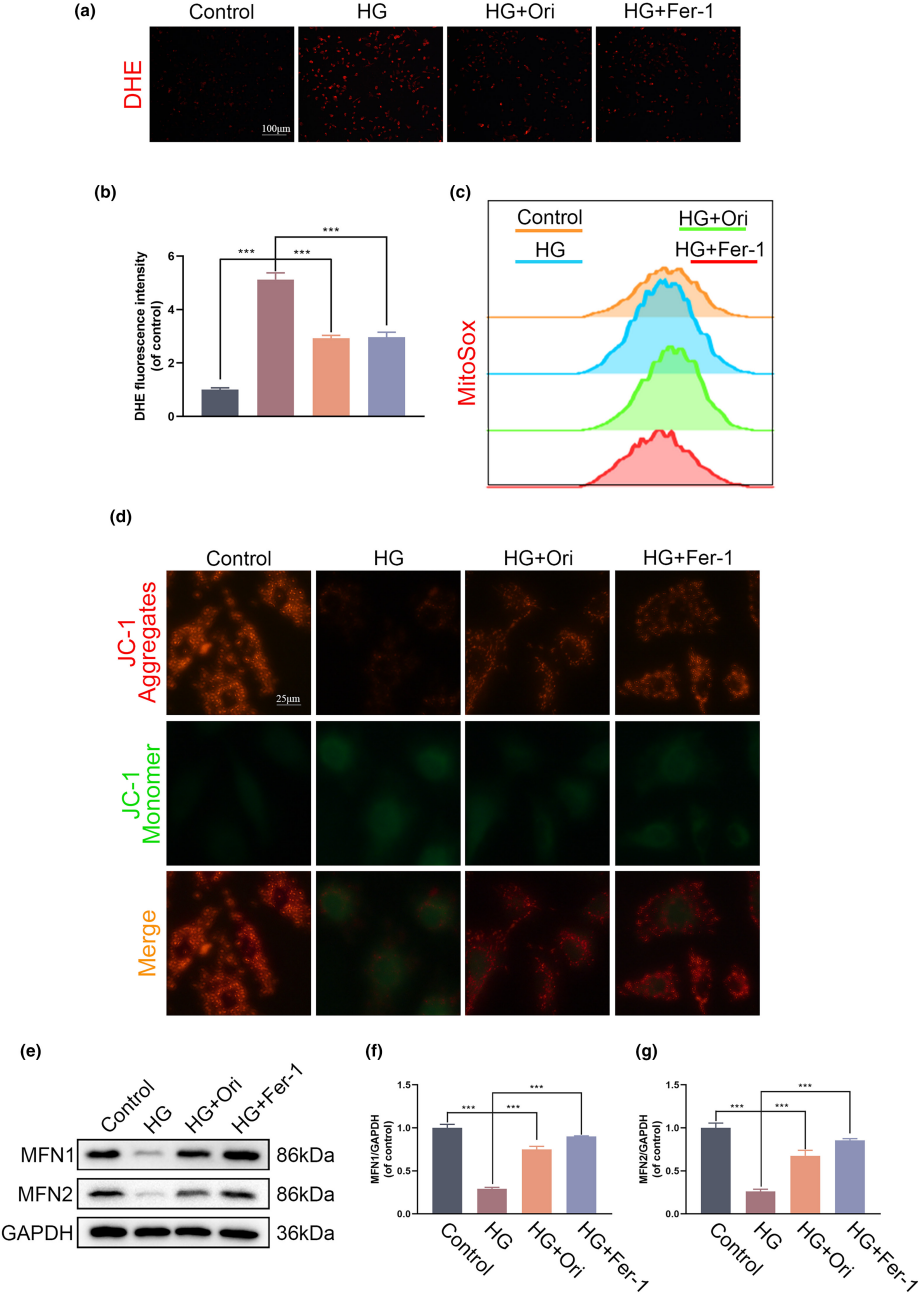
The mitochondrial dysfunction caused by HG in HUVECs can be reversed by treatment with Orientin. (a and b) Quantitative analysis of the DHE staining as well as representative images of each of the different groups. (c) The MitoSox (red) stain was used to conduct the MitoROS staining. The intensity of fluorescence as measured by flow cytometry in relation to its mean value. (d) Using confocal fluorescence microscopy, JC‐1 aggregates (shown in red) and monomers (shown in green) were found in HUVECs. (e–g) Western blot analysis comparing the levels of MFN1 and MFN2 expression in HUVECs. A comparison between the two groups is indicated by an asterisk. ***p < .001. All data are from *n* = 3 independent experiments.

The DHE fluorescent probe was utilized in order to determine the quantities of ROS that were present within the cells. It was demonstrated that the amounts of ROS in HUVECs decreased when Orientin and Fer‐1 were utilized after the administration of HG treatment (Figure 4a,b). Furthermore, mitochondrial reactive oxygen species (mtROS) were identified through the utilization of flow cytometry with the MitoSox probe. Following the introduction of HG, the findings indicated a significant increase in mtROS, which is indicative of the development of damage in the mitochondria that is induced by oxidative stress. Nevertheless, administration of Orientin and Fer‐1 effectively diminished the buildup of mtROS (Figure 4c). As an additional point of interest, the JC‐1 assay revealed that the intensity of green fluorescence increased, whereas the intensity of red fluorescence that was emitted decreased as a result of the stimulation of HG. Administration of Orientin and Fer‐1 effectively returned both fluorescence levels to a state that closely resembled the normal physiological levels (Figure 4d). It is necessary for the presence of a large number of proteins, one of which being mitofusins (MFN1/MFN2), in order for mitochondria to fuse together. The Western blot analysis showed that the HG group had lower levels of MFN1 and MFN2 expression than the other groups. The postconditioning with Orientin and Fer‐1 was successful in reducing the effects of the changes (Figure 4e–g). Based on the findings, it appears that Orientin was able to effectively combat the mitochondrial dysfunction that was brought about by HG.

### 3.5 Orientin promoted cell function in HG‐treated HUVECs

**FIGURE 5:**
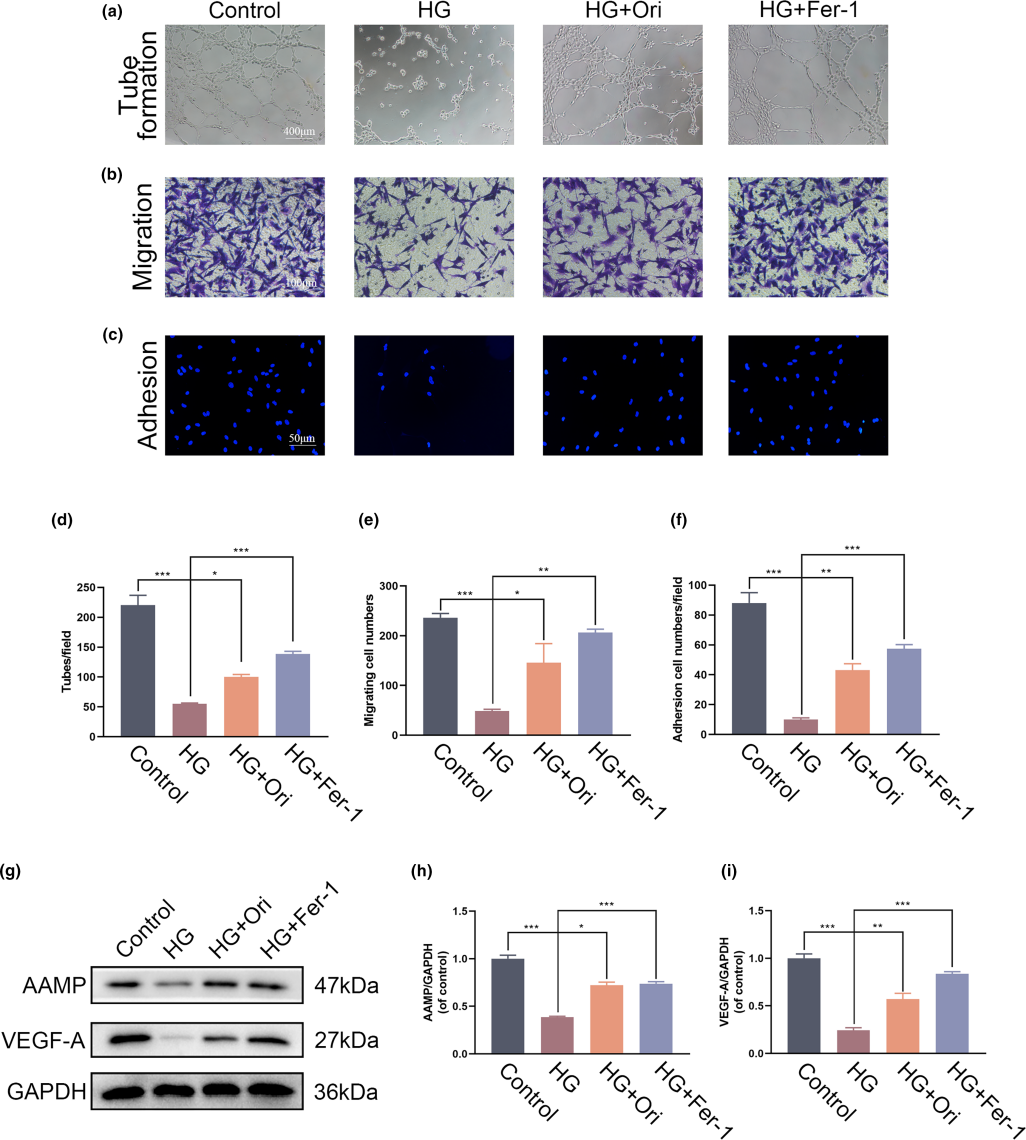
Orientin was able to restore cell function in HUVECs that had been treated with HG. (a and d) An evaluation of the Orientin‐mediated HUVECs neovascularization using the tube formation assay. (b and e) Using the transwell system, an evaluation of the migration of Orientin‐mediated HUVECs was performed. (c and f) The cell‐matrix adhesion assay was used to evaluate the effect of Orientin on HUVECs’ ability to adhere. A comparison between the two groups is indicated by an asterisk. (g‐i) WB analysis of the expression of AAMP and VEGF‐A in HUVECs. *p < .05; **p < .01; ***p < .001. All data are from *n* = 3 independent experiments.

In order to evaluate the possible therapeutic benefits of Orientin on cellular functions, a set of experiments were performed on HUVECs that had been subjected to HG therapy. An investigation into the neovascularization of HUVECs following Orientin therapy was carried out with the help of the tube formation evaluation. Following that, the number of structures that resembled capillaries that were present in each group was counted. The findings demonstrated that HG had a strong inhibitory effect on neovascularization in HUVECs. However, prior administration of Orientin and Fer‐1 provided protection against the harmful consequences of HG (Figure 5a,d). Following that, a transwell migration assay was conducted to examine the notion that Orientin facilitates the migration of HUVECs. The assessment was conducted on cells stained with hematoxylin that had migrated to the basolateral membrane. Statistical analysis revealed that the application of HG resulted in a notable reduction in the quantity of cells capable of migration. Nevertheless, the impacts were mitigated by the presence of Orientin and Fer‐1 (Figure 5b,e). In order to assess the influence of Orientin on the adherence of HUVECs, a fibronectin adhesion test was conducted as the concluding stage of the procedure. Statistical study demonstrated that HG stimulation had a considerable inhibitory effect on the amount of cells that adhered. Nevertheless, Orientin and Fer‐1 were found to effectively offset this effect (Figure 5c,f). The findings that were obtained from Western blot analysis were in agreement with the findings that were obtained from the angio‐associated migratory cell protein (AAMP), which is also known as a migration marker, and the vascular endothelial growth factor A (VEGF‐A), which is also known as an angiogenic factor. The protein levels of AAMP and VEGF‐A were shown to be lower in HUVECs that were grown under HG conditions, according to quantitative research that was carried out utilizing Western blots. However, the application of Orientin and Fer‐1 postconditioning was able to correct these alterations (Figure 5g–i).

### 3.6 Orientin protected HUVECs against ferroptosis via the Nrf2/GPX4 signaling pathway

**FIGURE 6:**
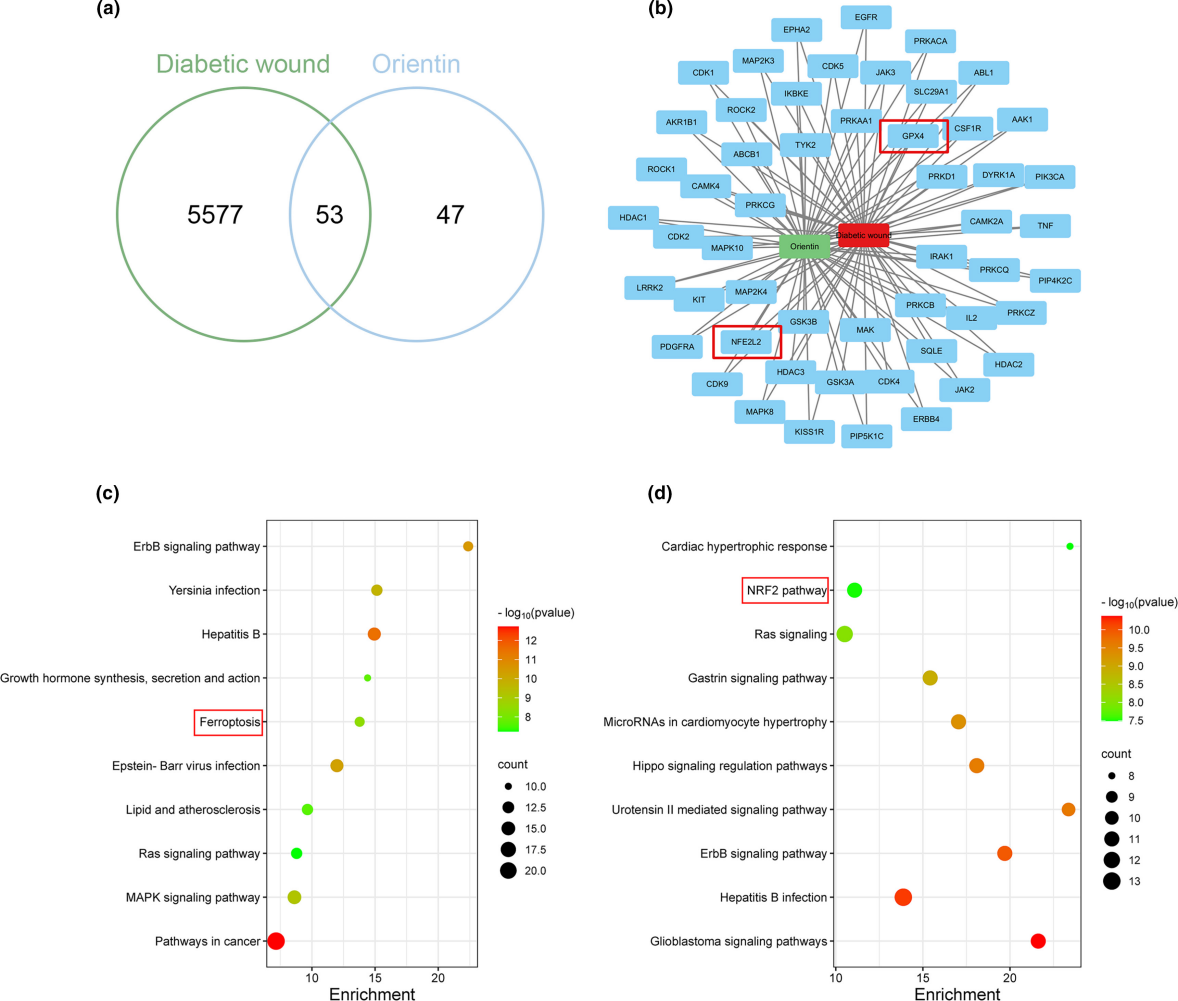
Network pharmacology analysis. (a) Venn diagram of Ori and diabetic wound targets. (b) The primary clusters extracted from the PPI network illustrate the main functional gene clusters of potential targets. (c) KEGG pathway of core targets in Ori against diabetic wound. (d) Wikipedia analysis of core targets in Ori against diabetic wound.

First, using network pharmacology analysis, this study investigated the mode of action of Ori in treating diabetic wound. A network consisting of 53 genes and 6630 edges was generated through the intersection of the DW and Ori networks (Figure 6a). Utilizing the MCODE technique within Cytoscape software (version 3.9.1), we successfully identified key gene targets, namely Nrf2 and GPX4 (Figure 6b). Furthermore, the KEGG pathway enrichment analysis revealed the enrichment of 80 pathways. Notably, the top 10 signaling pathways closely associated with Ori treatment of diabetic wound, as depicted in Figure 6c,d, include ferroptosis, mitogen‐activated protein kinase (MAPK) signaling pathway, pathways in cancer, lipid and atherosclerosis, Hepatitis B, and erythroblastic oncogene B (ErbB) signaling pathway. In addition, Nrf2 pathway is also enriched in Wikipedia analysis.

**FIGURE 7:**
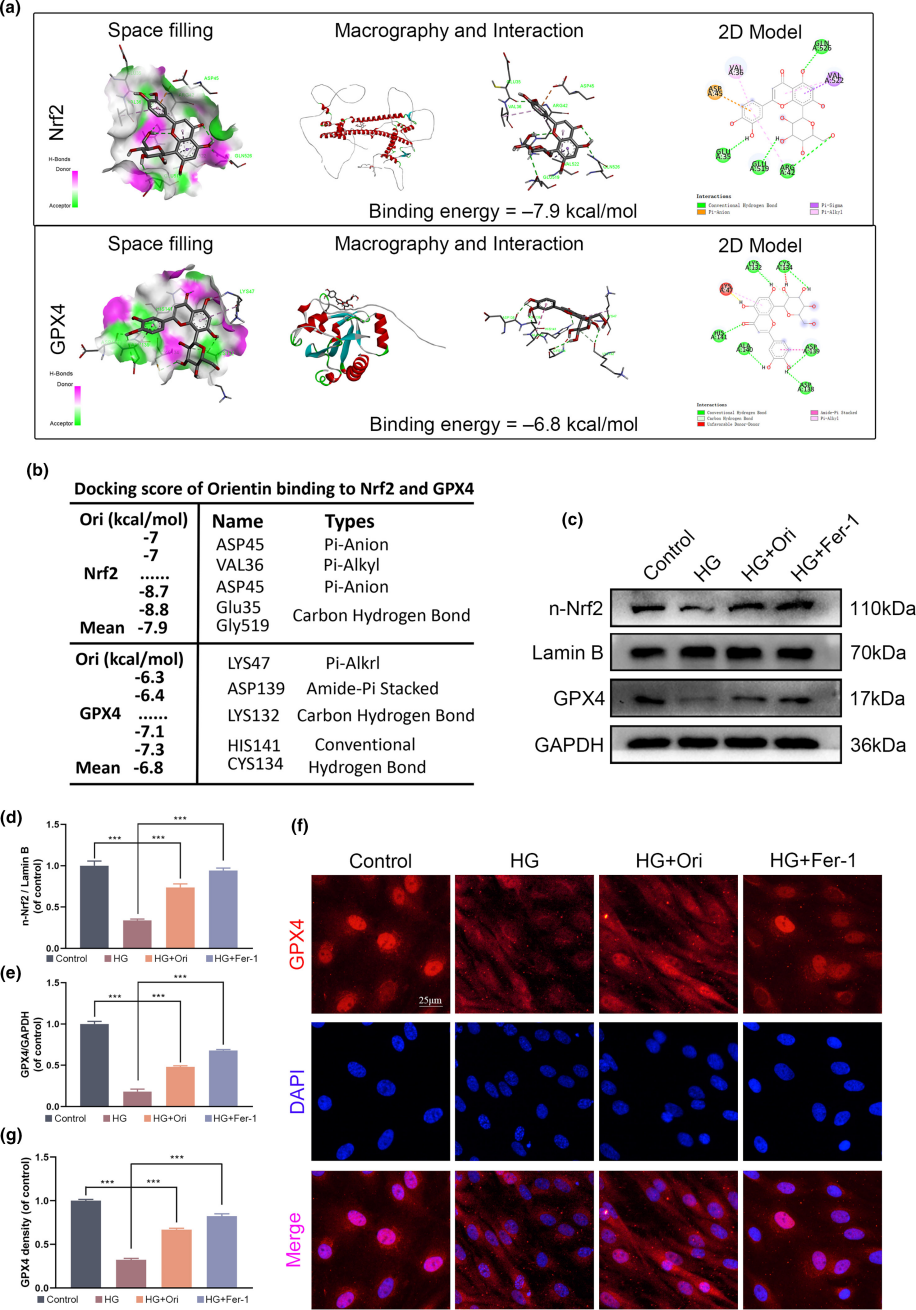
Orientin suppresses ferroptosis via activation of the Nrf2/GPX4 signaling pathway in vitro. (a and b) A 3D binding model is illustrated by using the ribbon model, which is applied to represent protein residues. When Orientin is docked with either Nrf2 or GPX4, the binding sites have affinities that are, respectively, −7.9 and −6.8 kcal/mol. In order to demonstrate the binding of Orientin in the Nrf2 and GPX4 pockets, a space‐filling model was utilized. (c–e) The expression of n‐Nrf2 and GPX4 in HUVECs was analyzed by Western blot. (f and g) Representative images and quantification analysis of GPX4 fluorescence of HUVECs. A comparison between the two groups is indicated by an asterisk. ***p < .001. All data are from *n* = 3 independent experiments.

Molecular docking simulations were conducted using artificial intelligence (AI)‐powered software to investigate the affinity between Orientin and proteins on the Nrf2/GPX4 axis. The chemical structure of Orientin was analyzed, and all possible models were tested to demonstrate its interaction and docking with the Nrf2/GPX4 docking site. Additionally, a space‐filling model was developed in order to provide additional evidence of the connection, and a ribbon model was deployed in order to highlight both the general and specific components of these interactions. A free energy of −7.9 and −6.8 kcal/mol, respectively, took place between Orientin and either Nrf2 or GPX4 (Figure 7a). Based on the molecular docking analysis, Orientin first creates a Pi‐Donor, then forms a Conventional Hydrogen Bond with ARG42, GLN526, GLU519, GLU35, and then a Pi‐Anion with ASP45, a Pi‐Sigma with VAL522 and a Pi‐Alkyl with VAL522, VAL36, and ARG42, and ultimately interacts with Nrf2. After that, it makes a Conventional Hydrogen Bond contact with HIS141, CYS134, LYS132, ALA140, ASP138, and ASP139, a Carbon Hydrogen Bond with LYS132, an Amide‐Pi Stacked with ASP139, ALA140, and ultimately interacts with GPX4. A significant link between the Orientin route and the Nrf2/GPX4 pathway has been found to exist, according to the results of the software simulation (Figure 7b). This finding was validated by conducting a Western blot study that is represented in the figure (Figure 7c–e). The results of this investigation revealed that the suppressive effect of HG on the movement of Nrf2 into the nucleus and the production of GPX4 could be successfully alleviated by administering either Orientin or Fer‐1. These findings were supported by the results of the GPX4 immunofluorescence staining experiment, which were in agreement with the findings themselves (Figure 7f).

**FIGURE 8:**
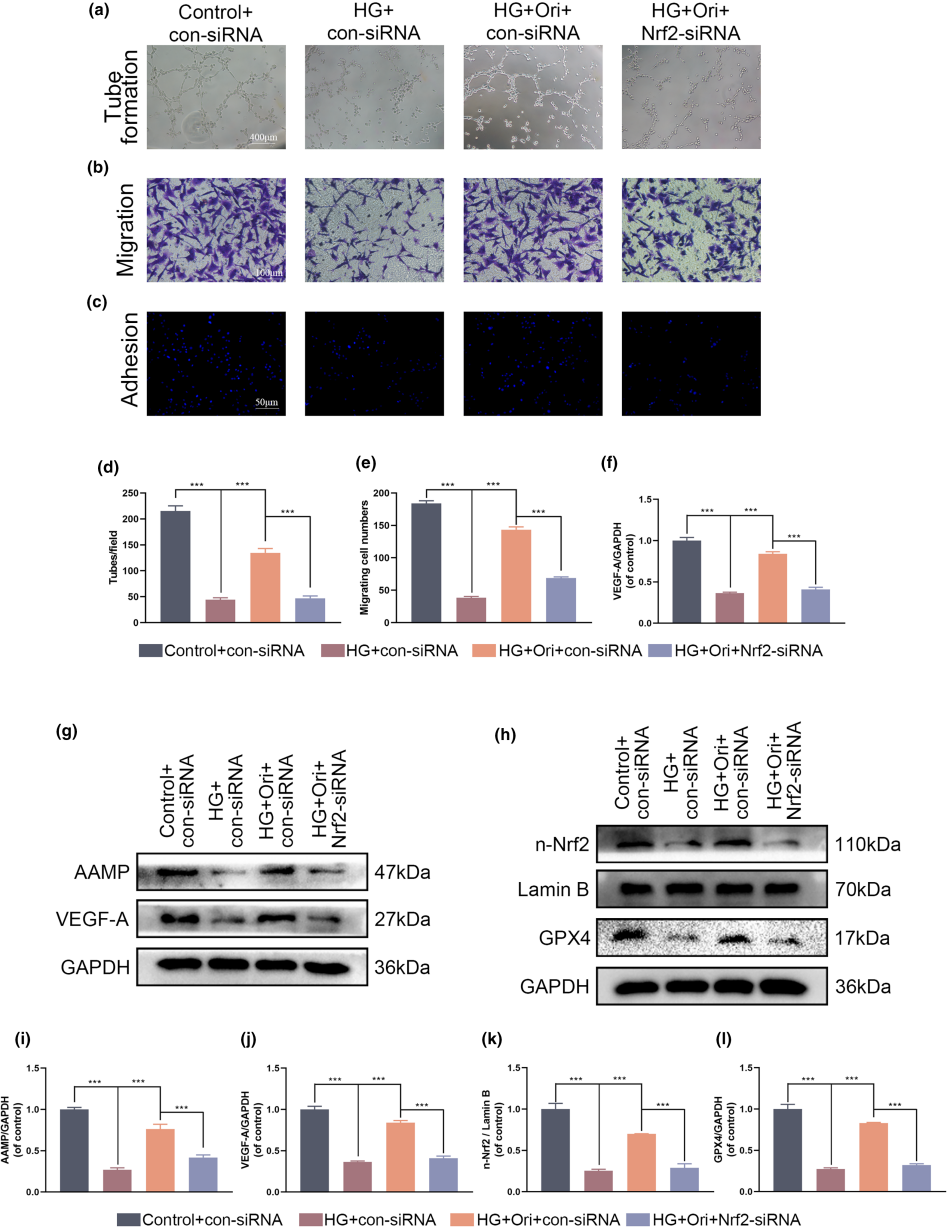
Inhibition of ferroptosis and improving HUVECs function by Orientin can be regulated by Nrf2–siRNA. (a and d) Evaluation of the neovascularization of HUVECs induced by Orientin using the tube formation assay in a variety of different groups. (b and e) Evaluation of the effect of Orientin on the migration of HUVECs using the transwell system in various groups. (c and f) Comparison of the levels of Orientin‐mediated HUVECs adhesion using a cell‐matrix adhesion assay in each of the different groups. (g and i) Western blot analysis of the expression of AAMP and VEGF‐A in HUVECs. (h and j) Western blot analysis of the expression of n‐Nrf2 and GPX4 in HUVECs. (k and l) Quantitative analysis of the expression of n‐Nrf2 and GPX4 in HUVECs. A comparison between the two groups is indicated by an asterisk. ***p < .001. All data are from *n* = 3 independent experiments.

It was determined that the Nrf2–siRNA was effective in inhibiting the expression of the Nrf2 gene, which brought to light the significance of the Nrf2 pathway in ferroptosis that was brought about by HG (Figure 8). The effects of Nrf2–siRNA were found in multiple cell function experiments, such as the tube formation assay, the transwell migration assay, and the fibronectin adhesion assay, resulting in a decrease in the activating effect of Orientin (Figure 8a–f). In addition, the stimulatory impact of Orientin on the Nrf2/GPX4 pathway was reduced as a result of the action of Nrf2‐siRNA (Figure 8h,k,l). When HG and Nrf2‐siRNA were administered together, the expression levels of AAMP and VEGF‐A were decreased (Figure 8g,i,j). In a word, the suppression of Nrf2 resulted in the elimination of the beneficial effects of Orientin.

### 3.7 Orientin promotes wound healing process in DM mice

**FIGURE 9:**
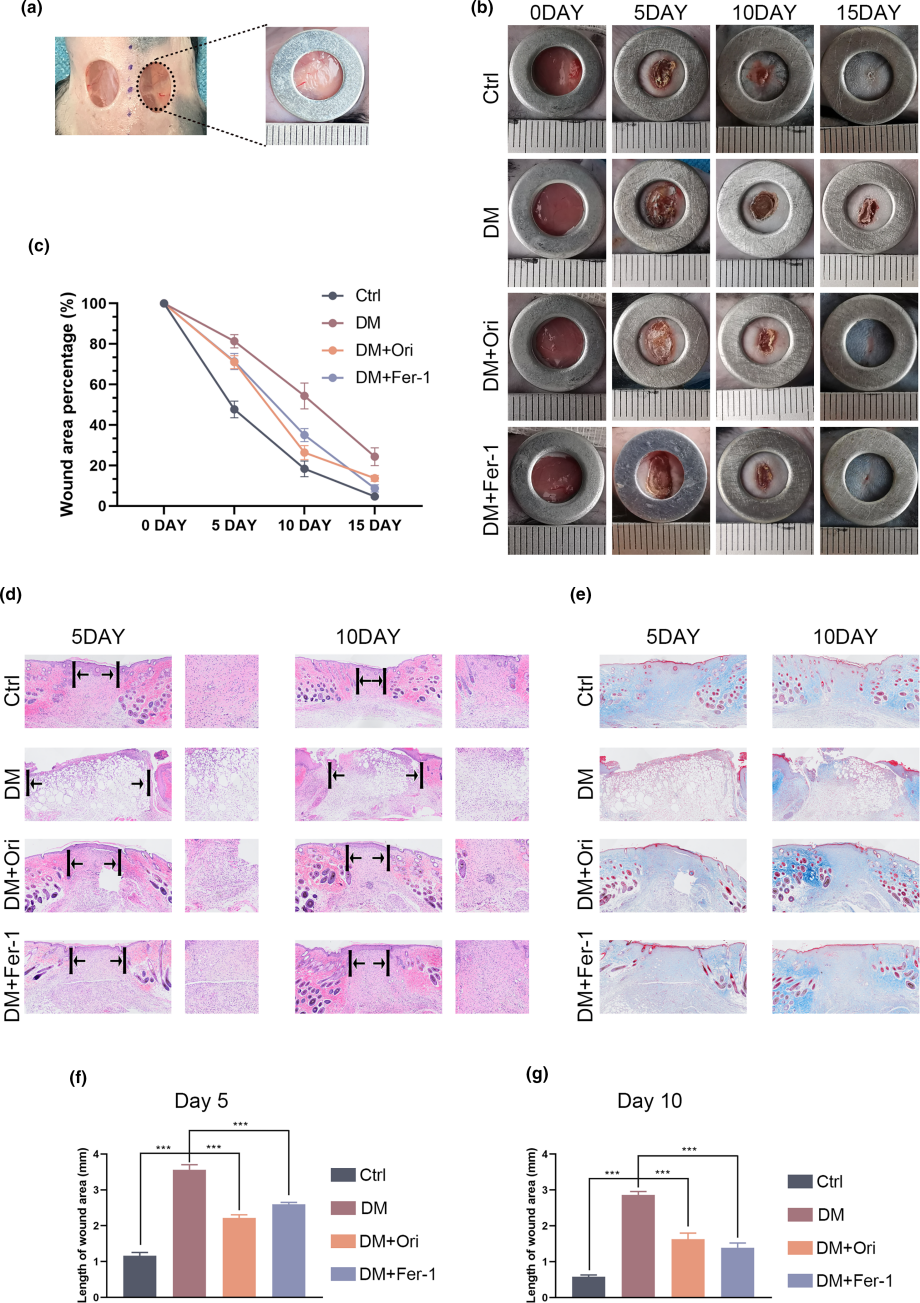
Orientin promotes diabetic wound healing process in mice. (a) Following the procedure described above, two round dermal wounds were created on opposite sides of the dorsal trunk of diabetic mice. (b and c) Representative digital images of wound closure for control and Orientin (20 mg/kg) group on day 0, day 5, day 10, and day 15. (d–g) H&E and Masson staining, as well as quantitative analysis, were performed on full‐thickness wounds on days 5 and 10. A comparison between the two groups is indicated by an asterisk. *p < .05; **p < .01; ***p < .001. All data are from *n* = 3 independent experiments.

To investigate the effectiveness of Orientin in healing diabetic wounds, we induced diabetes in mice via intraperitoneal injection of STZ, before creating skin wounds during surgery. Figure 9a depicts an image of a wound during the surgery, while it can be shown in Figure 9b that the group with diabetes had a slower rate of wound healing compared to the group that served as the control. Conversely, wound healing was expedited in diabetic mice treated with Orientin and Fer‐1 (Figure 9b,c). The three most significant pathological changes observed during the healing process were reepithelialization, granulation tissue, and wound length. The H&E staining of the tissue and the Masson staining were utilized in order to examine these changes. On days 5 and 10, the diabetic group did not display any obvious evidence of epithelialization, in contrast to the group that served as the control. However, diabetic wounds treated with Orientin and Fer‐1 showed more clear‐cut neoepidermis (Figure 9d–g).

### 3.8 Orientin enhanced angiogenesis and GPX4 expression in wound healing process in DM mice

**FIGURE 10:**
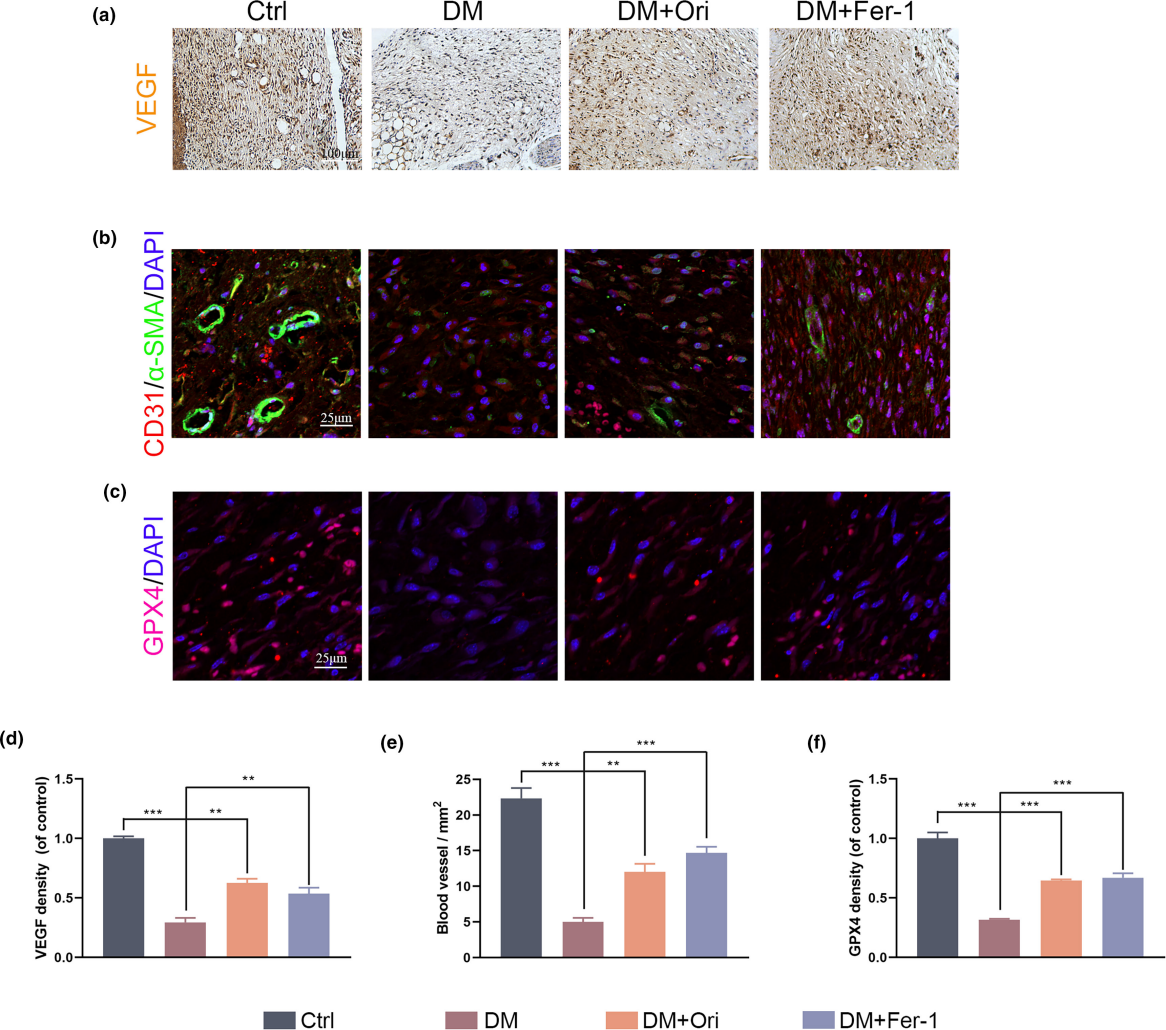
Orientin promoted an increase in angiogenesis and GPX4 expression during the healing process of diabetic wounds. (a and d) Representative images and quantitative analysis of the VEGF immunohistochemical staining were taken on day 10 after the operation. (b and e ) Images representative of the staining with immunofluorescence for CD31 and α‐SMA in vascular endothelial cells from each of the different groups, as well as quantitative analysis of the staining. (c and f) Images representative of the staining with immunofluorescence for GPX4 in vascular endothelial cells from each of the different groups, as well as quantitative analysis of the staining. A comparison between the two groups is indicated by an asterisk. *p < .05; **p < .01; ***p < .001. All data are from *n* = 3 independent experiments.

Experiments were conducted to determine whether Orientin significantly impacted the microvascular network on the backs of mice. Immunofluorescence and immunohistochemistry were used to investigate this. Diabetic mice treated with Orientin and Fer‐1 had a significantly higher number of VEGF‐positive vasculature than diabetic mice in the DM group (Figure 10a,d). Newly created and mature blood vessels were distinguished using SMA and CD31 immunofluorescence (IF) labeling, respectively, and there was a significant increase in the number of both in the dermal defect of the Orientin and Fer‐1 groups on day 10. In contrast, the DM group reduced the number of newly produced and mature blood vessels (Figure 10b,e). Additionally, the levels of GPX4 in the diabetic wounds that were treated showed a substantial rise when contrasted with the diabetic wounds that were treated with Orientin and Fer‐1 (Figure 10c,f). These findings suggest that Orientin acted as a catalyst for angiogenesis and played a substantial part in the improvement of diabetic wound healing.

## 4 DISCUSSION

Within the scope of this study, we utilized both in vitro and in vivo diabetes models to evaluate the effect that ferroptosis has on the healing process of diabetic wounds. The results of our in vitro investigations showed that high glucose levels led to decreased HUVECs survival and migration, as well as increased levels of ROS, lipid peroxidation products, and proteins related to ferroptosis on the part of the cells. However, treatment with either Ori or Fer‐1 was able to alleviate the negative effects of high glucose levels. Additionally, in a streptozotocin‐induced diabetes mouse model, diabetic mice treated with Ori or Fer‐1 experienced significantly faster wound healing rates. This was accompanied by decreases in ROS and ferroptosis levels, as well as an increase in the activation of the anti‐oxidation signaling pathway involving Nrf2/GPX4. Our findings suggest that Ori combats ROS, thereby inhibiting ferroptosis, and promotes the production of pro‐angiogenic proteins, ultimately leading to improved diabetic wound healing.

In cases of diabetic wounds, the continuous presence of high blood sugar levels can cause ongoing inflammation and an excessive buildup of ROS at the site of the wound. This, in turn, triggers a substantial rise in oxidative stress within the wound's surrounding environment, ultimately resulting in a delay in the healing process (Deng et al., 2023). Furthermore, Jin and Li et al. propose that HG‐induced ROS might trigger mitochondrial malfunction and increase ROS production, leading to death in cells through a process that relies on mitochondria (Jin et al., 2023; Li, Chen, et al., 2022). Hence, the control of ROS generation and the deterioration of mitochondrial activity in the HG milieu are pivotal elements in facilitating the healing of diabetic wounds. Iron excess has been detected in various chronic disorders as a result of aberrations in iron homeostasis (Dong et al., 2022). Savita et al. have documented that the occurrence of diabetic ferroptosis is pivotal in initiating inflammation in diabetic wounds (Khanna et al., 2010). Furthermore, Nancy S. and colleagues claim that oxidative stress and lipid peroxidation are widespread pathogenic processes that lead to both ferroptosis and delayed wound healing in diabetic ulcers (Younis et al., 2022). This is a proposition that has been published in the journal *Diabetes Care*. Additionally, prior studies have substantiated the effectiveness of another ferroptosis inhibitor, desferrioxamine (DFO), in enhancing the healing of diabetic wounds (Gao et al., 2018). A compacted mitochondrial membrane, decreased mitochondrial cristae, and a breakdown in the mitochondrial membrane are the cytological characteristics that distinguish ferroptosis from other types of mitochondrial dysfunction (Li, Wu, et al., 2022). Ferroptosis is the result of a decrease in mitochondrial bioenergetics and an induction of glutathione depletion, both of which ultimately lead to ferroptosis (He et al., 2023). For this reason, the restoration of typical mitochondrial function has the potential to serve as an effective therapeutic method for the treatment of ferroptosis condition.

There is a consensus among researchers that Nrf2 is an essential transcription factor that is responsible for promoting antioxidant activity. Increasing the expression of genes that encode a large number of antioxidant enzymes is how it accomplishes its function (Sun et al., 2017). Because it possesses the unique ability to convert phospholipid hydroperoxides into lipid alcohols, GPX4 is a crucial component in the process of guarding against ferroptosis (Wu et al., 2022). In contrast to the other isoforms of GPX, GPX4 is the only enzyme that has the potential to reduce the amount of phospholipid hydroperoxides found in cell membranes. By successfully eliminating intracellular lipid ROS, GPX4 also plays a significant role in preventing cellular ferroptosis, which is a condition that can be fatal (Jiang et al., 2020). The deactivation of GPX4 is the key component that leads to the accumulation of lipid peroxides, which ultimately leads to the beginning of the ferroptosis process. It has been demonstrated through research carried out by Ni et al. and Ren et al. that the activation of the Nrf2/GPX4 pathway has the ability to successfully prevent ferroptosis in mice that have suffered spinal cord injuries and in hippocampal neurons that have been subjected to radiation, respectively (Ni et al., 2023; Ren et al., 2023). The results offer strong evidence for the involvement of the Nrf2/GPX4 pathway in ferroptosis triggered by HG.

**FIGURE 11:**
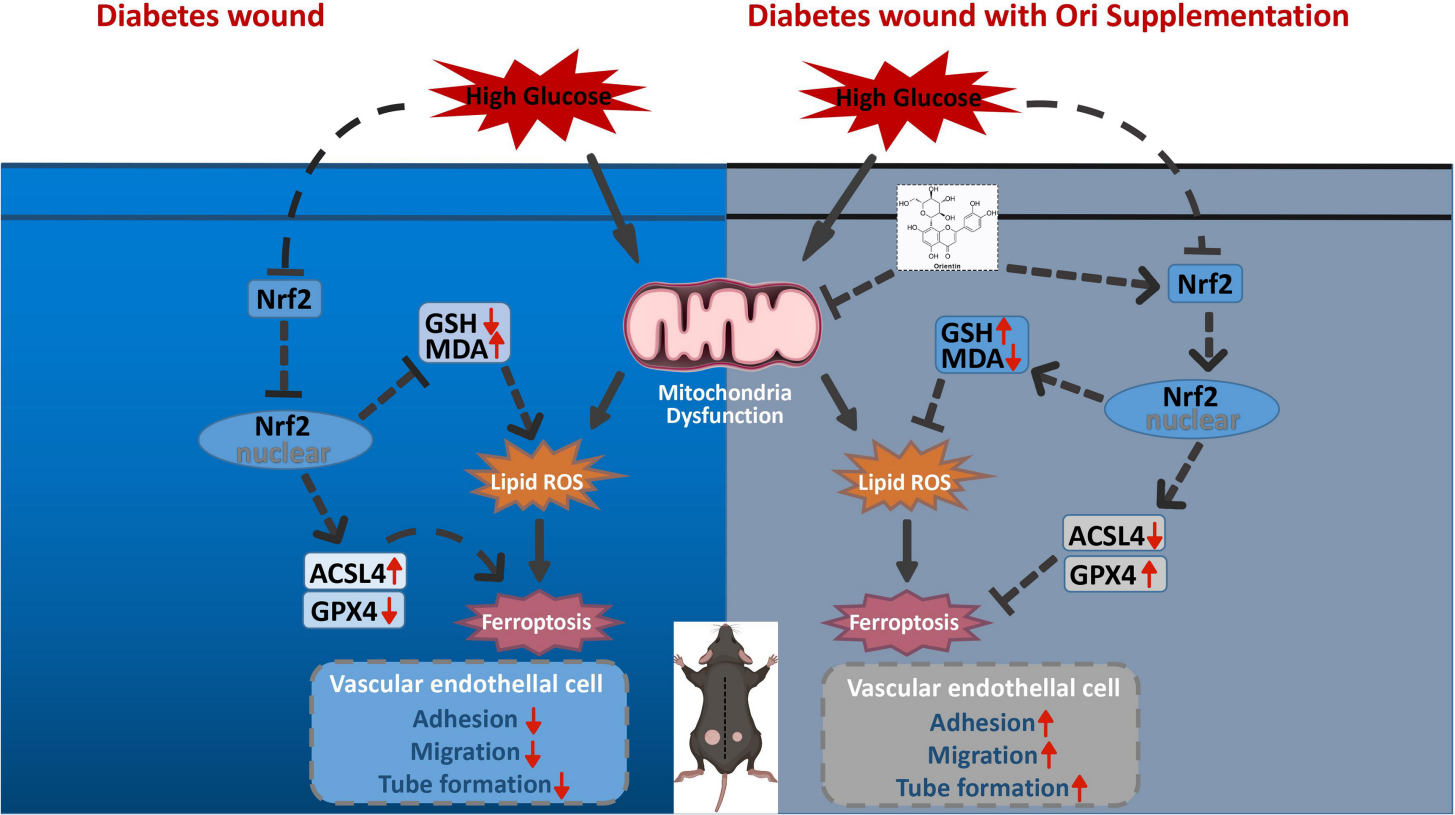
Mechanisms of action of Orientin on HUVECs that have been induced by HG. Orientin promotes diabetic wound healing by activating the Nrf2/GPX4 pathway. It also decreases HG‐induced ferroptosis by reducing the accumulation of ROS and rescuing mitochondrial dysfunction in HUVECs.

Orientin is a C‐glycosyl flavonoid that can be extracted from foods like rooibos tea and passion fruit. It possesses anti‐inflammatory, antitumor, and antioxidant activities (Tao et al., 2023). Research has shown that the Nrf2/GPX4 signaling pathway is highly associated to the process of healing damage caused by diabetes as well as mitochondria and ferroptosis. This is despite the fact that the precise method by which Orientin functions in diabetic wounds is not completely understood. By stimulating the Nrf2/GPX4 pathway in diabetic wound healing, we anticipated that Orientin may prevent HG from causing ferroptosis and restore mitochondrial dysfunction. This would be accomplished using Orientin. Ori appears to be responsible for the nuclear translocation of Nrf2, the expression of GPX4, and proteins that are associated with angiogenesis, according to the findings, as well as the adhesion and migration of HUVECs. In vivo investigations revealed that treatment with Ori can boost DM wound healing, angiogenesis, and the upregulation of GPX4 expression (Figure 11).

## 5 CONCLUSION

Ultimately, the data gained from our laboratory‐based and live organism examinations reveal that Orientin exhibits potential as a therapeutic focus for the process of repairing wounds in patients with diabetes. To be more specific, it appears that Orientin offers protective effects in HUVECs by enhancing the activity of mitochondria and reducing ferroptosis, which is triggered by high glucose levels. Furthermore, it is anticipated that the stimulation of the Nrf2/GPX4 signaling pathway may improve the process of wound healing in diabetic patients who are undergoing Orientin therapy.

## AUTHOR CONTRIBUTIONS

**Jia‐yi Yang:** Project administration (equal); writing – original draft (equal). **Chen Zhuang:** Project administration (equal); writing – original draft (equal). **Yu‐zhe Lin:** Formal analysis (equal); software (equal). **Yi‐tian Yu:** Methodology (equal); project administration (equal). **Chen‐cheng Zhou:** Resources (equal); visualization (equal). **Chao‐yang Zhang:** Formal analysis (equal). **Zi‐teng Zhu:** Investigation (equal); methodology (equal). **Cheng‐jie Qian:** Project administration (equal). **Yi‐nan Zhou:** Formal analysis (equal); software (equal). **Wen‐hao Zheng:** Data curation (equal); resources (equal). **Yu Zhao:** Conceptualization (equal); methodology (equal); resources (equal). **Chen Jin:** Conceptualization (equal); methodology (equal); resources (equal). **Zong‐yi Wu**: Conceptualization (equal); Funding acquisition (equal).

## CONFLICT OF INTEREST STATEMENT

The authors declare no conflicts of interest.

## Data Availability

The data that were utilized to support the findings of this study can be obtained from the author who corresponds to this work.
